# Identification of proliferative hepatocellular carcinoma using the SMARS score and implications for microwave ablation

**DOI:** 10.1186/s13244-024-01792-8

**Published:** 2024-09-10

**Authors:** Peng Zhou, Yan Bao, De-Hua Chang, Jun-Xiang Li, Tian-Zhi An, Ya-Ping Shen, Wen-Wu Cai, Lu Wen, Yu-Dong Xiao

**Affiliations:** 1 Department of Pathology, The Second Xiangya Hospital of Central South University, 410011 Changsha, China; 2 Department of Radiology, The Second Xiangya Hospital of Central South University, 410011 Changsha, China; 3 Institute of Radiology and Nuclear Medicine, Cantonal Hospital Lucerne, Spitalstrasse, CH-6000 Lucerne Switzerland; 4 Department of Interventional Radiology, Guizhou Medical University Affiliated Cancer Hospital, 550004 Guiyang, China; 5 Department of Interventional Radiology, The Affiliated Hospital of Guizhou Medical University, 550004 Guiyang, China; 6 Department of Liver Surgery, The Second Xiangya Hospital of Central South University, 410011 Changsha, China; 7 Department of Diagnostic Radiology, The Affiliated Cancer Hospital of Xiangya School of Medicine, Central South University, 410013 Changsha, China

**Keywords:** Hepatocellular carcinoma, Microwave ablation, Imaging feature, Prognosis

## Abstract

**Objective:**

To compare therapeutic outcomes of predicted proliferative and nonproliferative hepatocellular carcinoma (HCC) after microwave ablation (MWA) using a previously developed imaging-based predictive model, the SMARS score.

**Methods:**

This multicenter retrospective study included consecutive 635 patients with unresectable HCC who underwent MWA between August 2013 and September 2020. Patients were stratified into predicted proliferative and nonproliferative phenotypes according to the SMARS score. Overall survival (OS) and recurrence-free survival (RFS) were compared between the predicted proliferative and nonproliferative HCCs before and after propensity score matching (PSM). OS and RFS were also compared between the two groups in subgroups of tumor size smaller than 30 mm and tumor size 30–50 mm.

**Results:**

The SMARS score classified 127 and 508 patients into predicted proliferative and nonproliferative HCCs, respectively. The predicted proliferative HCCs exhibited worse RFS but equivalent OS when compared with nonproliferative HCCs before (*p* < 0.001 for RFS; *p* = 0.166 for OS) and after (*p* < 0.001 for RFS; *p* = 0.456 for OS) matching. Regarding subgroups of tumor size smaller than 30 mm (*p* = 0.098) and tumor size 30–50 mm (*p* = 0.680), the OSs were similar between the two groups. However, predicted proliferative HCCs had worse RFS compared to nonproliferative HCCs in the subgroup of tumor size 30–50 mm (*p* < 0.001), while the RFS did not differ in the subgroup of tumor size smaller than 30 mm (*p* = 0.141).

**Conclusion:**

Predicted proliferative HCCs have worse RFS than nonproliferative ones after MWA, especially in tumor size larger than 30 mm. However, the phenotype of the tumor may not affect the OS.

**Critical relevance statement:**

Before performing microwave ablation for hepatocellular carcinoma, the tumor phenotype should be considered because it may affect the therapeutic outcome.

**Key Points:**

- Proliferative hepatocellular carcinoma (HCC) may be identified using the SMARS score, an imaging-based predictive model.
- SMARS predicted proliferative HCCs have worse recurrence-free and equivalent overall survival compared to nonproliferative HCC after microwave ablation.
- Tumor phenotype should be considered before performing microwave ablation.

**Graphical Abstract:**

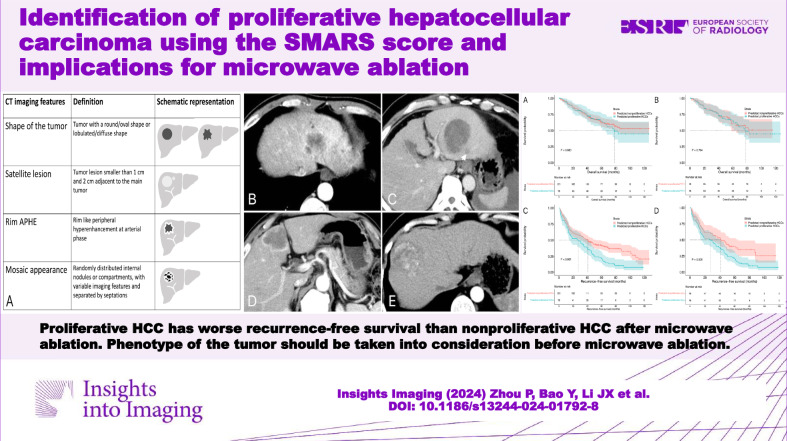

## Introduction

Hepatocellular carcinoma (HCC) is a highly heterogeneous disease at both the histological and molecular levels [1–3]. Recently, it was proposed that HCC could be divided into proliferative and nonproliferative phenotypes [4]. Previous studies have demonstrated that proliferative HCCs usually have a worse prognosis compared to nonproliferative HCCs after liver resection or transarterial chemoembolization [5, 6]. However, whether tumor phenotype correlates with the therapeutic outcomes of thermal ablation remains unclear. Thermal ablation, which includes radiofrequency ablation (RFA) and microwave ablation (MWA), is a curative treatment for patients with very early-stage or early-stage HCC who are not eligible for liver resection or transplantation [7–9]. RFA is the most widely used technique to date; however, MWA has been gradually gaining popularity over RFA owing to faster tissue heating and fewer heat sink effects [10]. One of the disadvantages of MWA is its limited efficacy for larger tumors and tumors located near major blood vessels [11]. Although several new techniques have been proposed to overcome the disadvantages of MWA [12, 13], local tumor recurrence after MWA is still very high. Consequently, the identification of other potential factors influencing local tumor control, such as the phenotype of the tumor, is crucial.

In the clinical setting, it is challenging to identify proliferative phenotype because the diagnosis of proliferative HCC is confirmed by histological samples and pretreatment biopsy is not routinely performed in HCC [14–17]. According to a previous study by Bao et al, proliferative HCCs may have unique imaging features [5]. They developed a computed tomography (CT) imaging feature-based predictive model, called the SMARS score, which has acceptable diagnostic performance for identifying proliferative phenotypes.

Therefore, based on the SMARS score, the present study aims to compare the overall survival (OS) and recurrence-free survival (RFS) between predicted proliferative and nonproliferative HCCs in patients treated with MWA.

## Materials and methods

### Study population

This multicenter retrospective study included five tertiary referral hospitals and was conducted in accordance with the Declaration of Helsinki. Institutional review board approvals were obtained from the participating hospitals, and the requirement for written informed consent was waived because of the retrospective study design.

Consecutive patients with unresectable very early-stage or early-stage HCC who underwent MWA as primary therapy between August 2013 and September 2020 were included. The inclusion criteria were as follows: (1) a single tumor of 50 mm or less, or 2–3 tumors that were each smaller than 30 mm (considering the indication of MWA), (2) ECOG performance status (PS) of 0, (3) albumin-bilirubin (ALBI) score of I or II without moderate to massive ascites, and (4) absence of macrovascular invasion or extrahepatic metastasis. The exclusion criteria were as follows: (1) absence of baseline CT imaging 1 month prior to MWA, (2) poor imaging quality, (3) absence of baseline laboratory information within a week prior to MWA, and (4) absence of follow-up data.

### Imaging acquisition

The CT examination was performed on four different machines using the following parameters: (1) Aquilion One scanner (Canon Medical Systems): tube voltage, 120 kVp; tube current, auto; rotation time, 0.5 s; matrix, 512 × 512; field of view, 400 × 400 mm; and slice thickness, 5 mm; (2) Somatom Definition As+ scanner (Siemens): tube voltage, 120 kVp; tube current, auto; rotation time, 0.5 s; matrix, 256 × 256; field of view, 452 × 452 mm; and slice thickness, 5 mm; and (3) Somatom Force scanner (Siemens): tube voltage, 110 kVp; tube current, auto; rotation time, 0.5 s; matrix, 512 × 512; field of view, 400 × 400 mm; and slice thickness, 5 mm; (4) Aquilion Prime scanner (Canon Medical Systems): tube voltage, 120 kVp; tube current, 300 mAs; rotation time, 0.35 s; matrix, 256 × 256; field of view, 400 × 400 mm; and slice thickness, 4 mm.

### Data collection and imaging analysis

Data on clinical variables were collected, including age, sex, etiology of the underlying liver disease, and liver cirrhosis. Imaging parameters included the number of tumors, tumor size, shape of the tumor, mosaic architecture, rim arterial phase hyperenhancement (APHE), and satellite lesions. Laboratory parameters included neutrophil count, lymphocyte count, platelet count, serum albumin, total bilirubin, and alpha-fetoprotein (AFP).

The CT images were independently reviewed by two board-certified radiologists with 13 and 18 years of experience in abdominal imaging, and the corresponding CT imaging features were recorded accordingly. For patients with multiple tumors, imaging features of the largest tumor were recorded. In cases of disagreement between the two radiologists, a final decision was made by consensus. The SMARS score was calculated as described previously, which included five parameters, such as **S**hape of tumor, **M**osaic architecture, **A**FP level, **R**im APHE, and **S**atellite lesion [5]. Briefly, 0.767 × Shape of tumor + 1.196 × Mosaic architecture + 0.881 × AFP level + 2.506 × Rim APHE + 1.178 × Satellite lesion − 8.811. A cutoff value of −0.49 was used to identify predicted proliferative and nonproliferative HCCs.

### Treatment approach and follow-up

The treatment approach was discussed by a tumor board that included surgeons, interventional radiologists, oncologists, diagnostic radiologists, and hepatologists. Clinicians discussed the treatment recommendations with the patients, and a final decision was made by consensus. The MWA procedures (KY-2000, Jiangsu Kangyou Medical Instrument; ECO-100AI10, Nanjing ECO Medical Technology) were performed by several board-certified senior interventional radiologists. Under CT guidance, the antenna was inserted percutaneously into the tumor. An overlapping technique was used for tumors larger than 30 mm. In patients with multiple tumors, ablation was performed for all tumors in a single session. The MWA was set at 60–140 W, and the ablation time was 3–25 min. Intraprocedural contrast-enhanced CT was performed to determine the safety margins. The technical success of ablation was defined as the complete ablation of the tumor with a safety margin of at least 0.5 cm on CT images.

Patients were observed for 2–3 months after MWA and at least every 6 months thereafter. Contrast-enhanced CT or magnetic resonance imaging, and determination of serum AFP levels were routinely performed to monitor recurrence. Follow-up was performed via telephone interviews (March 2024) or during the last visit to the hospital if a telephone interview was unavailable. The primary endpoint was OS, defined as the time interval between the date of MWA and the date of death or last follow-up. The secondary endpoint was RFS, defined as the time interval between the date of MWA and the date of recurrence or last follow-up.

### Subgroup analysis

To compare the therapeutic outcomes of predicted proliferative and nonproliferative HCCs in the subgroups, patients were further divided into groups according to tumor size: smaller than 30 mm and 30–50 mm.

### Statistical analysis

Continuous variables are presented as the mean ± standard deviation (SD) or the median with interquartile range (IQR). Categorical variables are presented as numbers with percentages. Categorical variables were compared using the χ2 test or Fisher’s exact test, as appropriate. Continuous variables were compared using the Mann‒Whitney *U*-test or *t*-test, as appropriate. The inter-reader agreement between the two radiologists regarding the CT imaging features in the SMARS score was calculated using the kappa coefficient. Based on the SMARS scores assigned to each patient, the entire study population was divided into predicted proliferative and nonproliferative HCCs, respectively. Propensity score matching (PSM) analysis was applied for baseline characteristics with statistical significance between the two groups to reduce potential confounding and selection biases. The optimal caliper for PSM was set to 0.1. The RFS and OS were compared between the two groups using the log-rank test before and after matching. Statistical analyses were performed using R software version 4.0.2 (R Foundation for Statistical Computing; http://www.R-project.org), and a two-sided *p* < 0.05 denoted statistical significance.

## Results

### Baseline characteristics of the patients

**Fig. 1:**
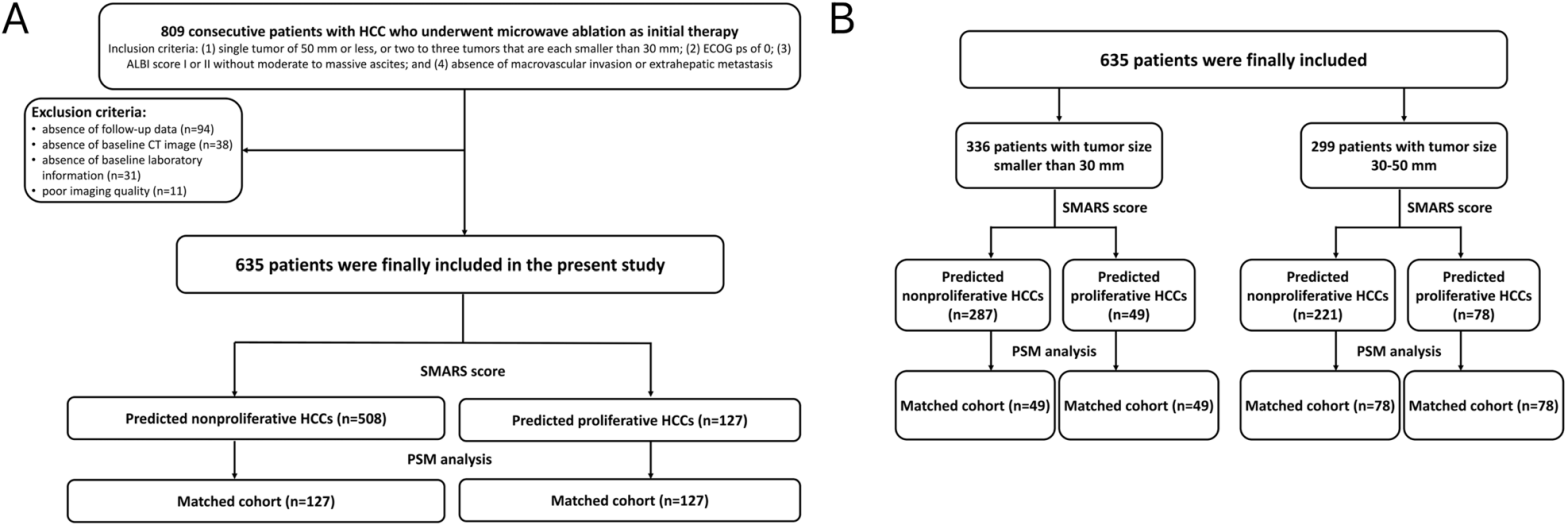
Flowchart of the study. Patients in the entire study population (**A**). Patients in the subgroups of tumor size smaller than 30 mm and tumor size 30–50 mm (**B**)

The entire study population included 635 patients (540 males and 95 females, with a mean age of 53.8 ± 11.4 years). A flowchart of the study population is shown in Fig. 1. The diagnosis of HCC was based on typical imaging features according to the LI-RADS criteria (*n* = 493) or pathology if the imaging features did not fulfill the LI-RADS criteria (*n* = 142). The baseline characteristics of the patients are summarized in Table 1. The median follow-up time was 49.2 months (IQR: 31.5–68.7 months). By the end of the follow-up period, 235 patients had died (37.0%, 235/635) and 415 had recurrence (65.4%, 415/635). The 1-, 2-, 3-, 4-, and 5-year OS rates were 92.7%, 84.3%, 75.8%, 68.4%, and 63.2%, while the 1-, 2-, 3-, 4-, and 5-year RFS rates were 75.3%, 61.5%, 51.1%, 42.2%, and 37.7%, respectively. The median OS for the entire study population was not reached, while the median RFS was 37.9 months (95% CI: 32.5–43.2 months).

**Table 1:** Baseline characteristics of patients in the entire study population

Baseline characteristics	
Age (years)*	53.8 ± 11.4
Gender (male, %)	540 (85.0)
Etiologies of liver diseases (*N*, %)	
Absence	101 (15.9)
HBV	485 (76.4)
Others	49 (7.7)
Liver cirrhosis (presence, %)	600 (94.5)
BCLC stage (*N*, %)	
0	23 (3.6)
A	612 (96.4)
Neutrophil (× 10^9^, IQR)^#^	2.86 (2.00–3.68)
Lymphocyte (× 10^9^, IQR)^#^	1.32 (1.02–1.75)
Platelet (× 10^9^, IQR)^#^	126.0 (82.0–172.0)
Albumin (g/L, IQR)^#^	39.6 (36.6–42.7)
Total bilirubin (µmol/L, IQR)^#^	15.2 (11.1–20.9)
ALBI (*N*, %)	
I	323 (50.9)
II	312 (49.1)
Satellite lesion (presence, %)	39 (6.1)
Tumor shape (lobulated, %)	150 (23.6)
Mosaic architecture (presence, %)	72 (11.3)
Rim APHE (presence, %)	79 (12.4)
AFP (> 200 ng/mL, %)	212 (33.4)
Tumor size (*N*, %)	
≤ 30 mm	336 (52.9)
30–50 mm	299 (47.1)
Tumor numbers (*N*, %)	
Solitary	357 (56.2)
2–3	278 (43.8)
Follow-up duration (months, IQR)^#^	49.2 (31.5–68.7)
1-year OS rate (%) (number of deaths)	92.7 (46)
2-year OS rate (%) (number of deaths)	84.3 (98)
3-year OS rate (%) (number of deaths)	75.8 (149)
4-year OS rate (%) (number of deaths)	68.4 (190)
5-year OS rate (%) (number of deaths)	63.2 (210)
Median OS (months, 95% CI)	Not reached
1-year RFS rate (%) (number of recurrences)	75.3 (157)
2-year RFS rate (%) (number of recurrences)	61.5 (244)
3-year RFS rate (%) (number of recurrences)	51.1 (309)
4-year RFS rate (%) (number of recurrences)	42.2 (360)
5-year RFS rate (%) (number of recurrences)	37.7 (380)
Median RFS (months, 95% CI)	37.9 (32.5–43.2)

*HBV* hepatitis B virus, *BCLC* Barcelona Clinic Liver Cancer, *ALBI* albumin-bilirubin, *APHE* arterial phase hyperenhancement, *AFP* alpha-fetoprotein, *OS* overall survival, *RFS* recurrence-free survival

* Data are presented as mean ± SD

^#^ Data are presented as median and IQR

### CT imaging features and the SMARS score

**Fig. 2:**
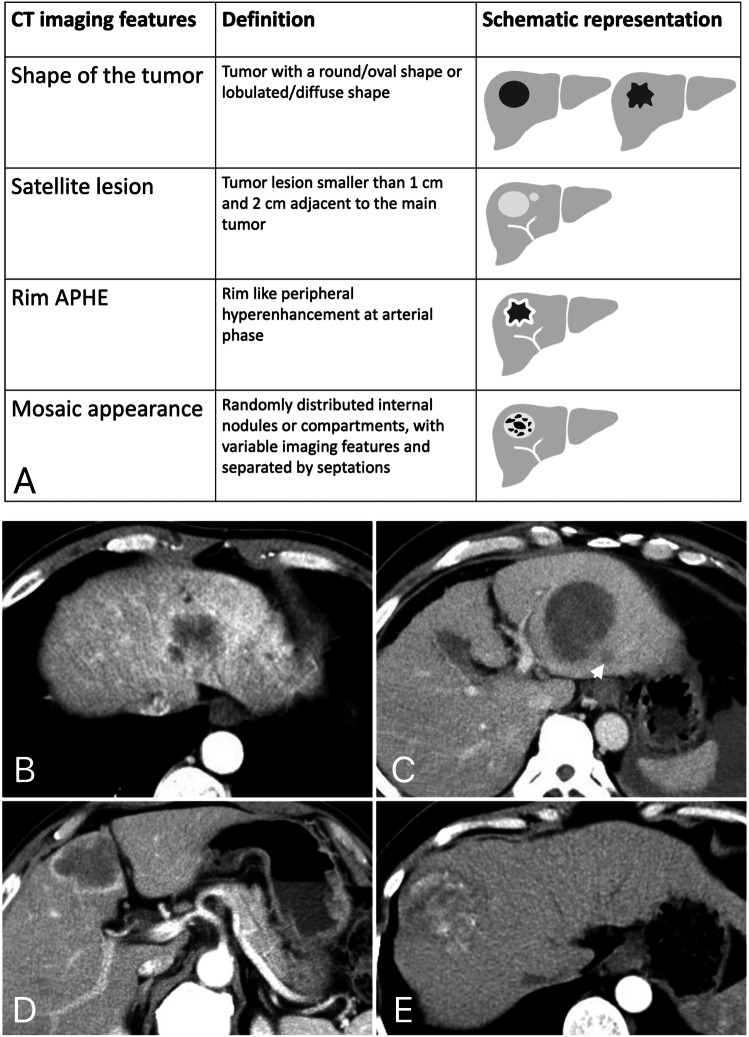
Illustration of the computed tomography imaging features (**A**) tumor with 39 mm of diameter and lobulated shape (**B**) tumor with 49 mm of diameter and satellite lesion (white arrow) (**C**) tumor with 45 mm of diameter and rim APHE (**D**) and tumor with 48 mm of diameter and mosaic architecture (**E**)

The inter-reader agreements for the CT imaging features were excellent (Kappa 0.84–0.91). A detailed illustration of the imaging features is shown in Fig. 2. Within the entire study population, 150 patients had a lobulated tumor shape (23.6%, 150/635), 39 patients had satellite lesions (6.1%, 39/635), 79 patients had rim APHE (12.4%, 79/635), 72 patients had mosaic architecture (11.3%, 72/635), and 212 patients had AFP levels greater than 200 ng/mL (33.4%, 212/635). Based on the SMARS scores, 127 patients were categorized as having predicted proliferative HCCs, and 508 patients were categorized as having predicted nonproliferative HCCs.

### Therapeutic outcomes of the two groups before and after matching in the entire study population

The median OS in both the predicted nonproliferative and proliferative HCCs was not reached, and there was no difference in OS between the two groups (*p* = 0.166). The median RFSs were 41.0 months (95% CI: 33.7–48.3 months) and 29.1 months (95% CI: 18.2–39.9 months) in predicted nonproliferative and proliferative HCCs groups, respectively, and the RFS was significantly better in nonproliferative HCCs than that of predicted proliferative ones (*p* < 0.001).

When compared with patients in the predicted nonproliferative HCCs, patients in predicted proliferative HCCs had higher neutrophil (*p* = 0.003) and platelet counts (*p* = 0.021), and more frequently tumor size greater than 30 mm (*p* < 0.001). Therefore, a PSM analysis was performed using those three parameters (neutrophil and platelet counts, and tumor size) to reduce potential confounding and selection bias. In total, 127 pairs of patients were matched accordingly. The baseline characteristics of the patients in the two groups before and after matching are summarized in Table 2.

**Table 2:** Baseline characteristics between predicted nonproliferative and proliferative HCCs in the entire study population

	Before matching			After matching		
	Nonproliferative (*n* = 508)	Proliferative (*n* = 127)	*p* value	Nonproliferative (*n* = 127)	Proliferative (*n* = 127)	*p* value
Age (years)*	53.8 ± 11.6	53.8 ± 10.8	0.989	55.3 ± 10.7	53.8 ± 10.8	0.257
Gender (*N*, %)			0.578			1.000
Male	434 (85.4)	106 (83.5)		106 (83.5)	106 (83.5)	
Female	74 (14.6)	21 (16.5)		21 (16.5)	21 (16.5)	
Liver cirrhosis (*N*, %)			0.664			0.582
Absence	27 (5.3)	8 (6.3)		6 (4.7)	8 (6.3)	
Presence	481 (94.7)	119 (93.7)		121 (95.3)	119 (93.7)	
Etiologies of hepatitis (*N*, %)			0.941			0.789
None	80 (15.7)	21 (16.5)		20 (15.7)	21 (16.5)	
HBV	388 (76.4)	97 (76.4)		95 (74.8)	97 (76.4)	
Others	40 (7.9)	9 (7.1)		12 (9.4)	9 (7.1)	
Tumor size (*N*, %)			< 0.001			0.897
≤ 30 mm	287 (56.5)	49 (38.6)		48 (37.8)	49 (38.6)	
30–50 mm	221 (43.5)	78 (61.4)		79 (62.2)	78 (61.4)	
Tumor numbers (*N*, %)			0.055			0.105
Solitary	276 (54.3)	81 (63.8)		93 (73.2)	81 (63.8)	
2–3	232 (45.7)	46 (36.2)		34 (26.8)	46 (36.2)	
Neutrophil (× 10^9^, IQR)^#^	2.74 (1.96–3.58)	3.18 (2.26–4.04)	0.003	3.05 (2.27–3.89)	3.18 (2.26–4.04)	0.681
Lymphocyte (× 10^9^, IQR)^#^	1.33 (1.00–1.74)	1.27 (1.04–1.89)	0.863	1.36 (1.06–1.86)	1.27 (1.04–1.89)	0.522
Platelet (× 10^9^, IQR)^#^	122.0 (81.0–167.0)	137.0 (97.0–185.0)	0.021	128.0 (94.0–180.0)	137.0 (97.0–185.0)	0.380
Albumin (g/L, IQR)	39.6 (36.7–42.7)	39.2 (36.5–43.2)	0.828	39.8 (36.7–42.6)	39.2 (36.5–43.2)	0.663
Total bilirubin (µmol/L, IQR)	14.9 (11.0–20.5)	16.0 (11.6–21.8)	0.445	16.1 (11.9–21.7)	16.0 (11.6–21.8)	0.731

*HBV* hepatitis B virus

* Data are presented as mean ± SD

^#^ Data are presented as median and IQR

**Fig. 3:**
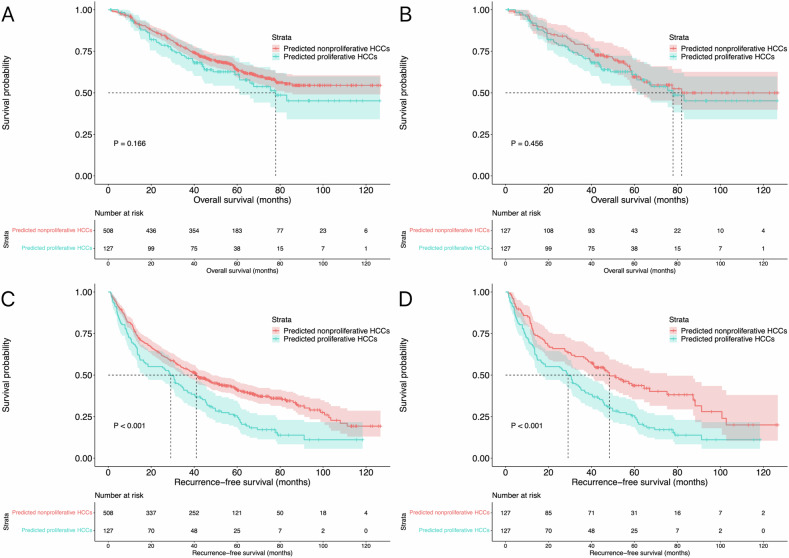
Survival curves of the entire study population. The OS of predicted nonproliferative and proliferative HCCs before matching (**A**) and after PSM (**B**) analysis. The RFS of predicted nonproliferative and proliferative HCCs before matching (**C**) and after PSM (**D**) analysis

After matching, the median OS was also not reached and there was no difference in OS between the two groups (*p* = 0.456). Regarding the median RFS, it was also better (*p* < 0.001) in patients with predicated nonproliferative HCCs (48.3 months; 95% CI: 34.3–62.3 months) when compared with that of the proliferative HCCs (29.1 months; 95% CI: 18.2–39.9 months). The survival curves before and after matching are shown in Fig. 3.

### Therapeutic outcomes of patients between the two groups in the subgroup of tumor size smaller than 30 mm

**Fig. 4:**
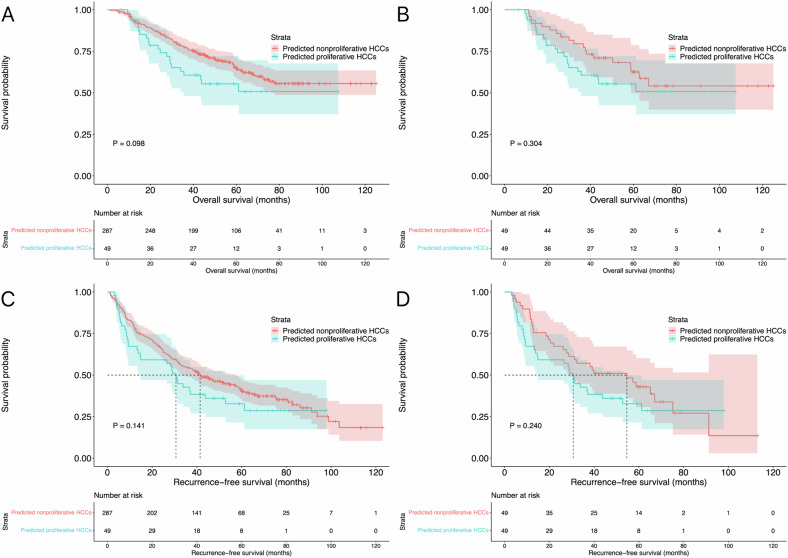
Survival curves in the subgroup of tumor size smaller than 30 mm. The OS of predicted nonproliferative and proliferative HCCs before matching (**A**) and after PSM (**B**) analysis. The RFS of predicted nonproliferative and proliferative HCCs before matching (**C**) and after PSM (**D**) analysis

In total, 287 and 49 patients had tumor size smaller than 30 mm in predicted nonproliferative and proliferative HCCs before matching, respectively. The OS (*p* = 0.098; median OS was not reached) and RFS (*p* = 0.141; 41.4 months, 95% CI: 30.9–52.0 months vs. 30.6 months, 95% CI: 26.6–34.6 months, respectively) did not significantly differ between the two groups. PSM analysis was performed to reduce selection bias, and 49 pairs were matched accordingly. The baseline characteristics of patients between the two groups before and after matching are summarized in Table 3. There were also no differences in OS (*p* = 0.304; median OS was not reached) and RFS (*p* = 0.240; 54.6 months, 95% CI: 30.4–78.8 months vs. 30.6 months, 95% CI: 26.6–34.6 months, respectively) between the two groups. The survival curves of patients in the subgroup of tumor size smaller than 30 mm before and after matching are shown in Fig. 4.

**Table 3:** Baseline characteristics between predicted nonproliferative and proliferative HCCs in the subgroup of tumor size smaller than 30 mm

	Before matching			After matching		
	Nonproliferative (*n* = 287)	Proliferative (*n* = 49)	*p* value	Nonproliferative (*n* = 49)	Proliferative (*n* = 49)	*p* value
Age (years)*	53.2 ± 11.6	52.9 ± 10.9	0.859	53.3 ± 10.5	52.9 ± 10.9	0.836
Gender (*N*, %)			0.757			0.779
Male	241(84.0)	42 (85.7)		41 (83.7)	42 (85.7)	
Female	46 (16.0)	7 (14.3)		8 (16.3)	7 (14.3)	
Liver cirrhosis (*N*, %)			0.084			0.715
Absence	12 (4.2)	5 (10.2)		3 (6.1)	5 (10.2)	
Presence	275 (95.8)	44 (89.8)		46 (93.9)	44 (89.8)	
Etiologies of hepatitis (*N*, %)			0.672			0.735
None	45 (15.7)	7 (14.3)		9 (18.4)	7 (14.3)	
HBV	222 (77.4)	37 (75.5)		37 (75.5)	37 (75.5)	
Others	20 (7.0)	5 (10.2)		3 (6.1)	5 (10.2)	
Tumor numbers (*N*, %)			0.026			1.000
Solitary	55 (19.2)	3 (6.1)		3 (6.1)	3 (6.1)	
2–3	232 (80.8)	46 (93.9)		46 (93.9)	46 (93.9)	
Neutrophil (× 10^9^, IQR)^#^	2.54 (1.79–3.47)	2.60 (1.90–3.79)	0.243	3.01 (2.03–3.63)	2.60 (1.90–3.79)	0.774
Lymphocyte (× 10^9^, IQR)^#^	1.31 (0.98–1.73)	1.27 (1.04–1.85)	0.765	1.29 (1.03–1.75)	1.27 (1.04–1.85)	0.977
Platelet (× 10^9^, IQR)^#^	116.0 (73.0–160.0)	124.0 (91.0–181.0)	0.102	119.0 (81.0–191.0)	124.0 (91.0–181.0)	0.793
Albumin (g/L, IQR)	39.8 (36.7–43.1)	40.7 (36.8–44.1)	0.436	40.8 (36.1–43.4)	40.7 (36.8–44.1)	0.969
Total bilirubin (µmol/L, IQR)	15.0 (11.3–21.7)	18.0 (13.1–22.6)	0.123	16.3 (13.2–26.7)	18.0 (13.1–22.6)	0.898

*HBV* hepatitis B virus

* Data are presented as mean ± SD

^#^ Data are presented as median and IQR

### Therapeutic outcomes of patients between the two groups in the subgroup of tumors size 30–50 mm

**Fig. 5:**
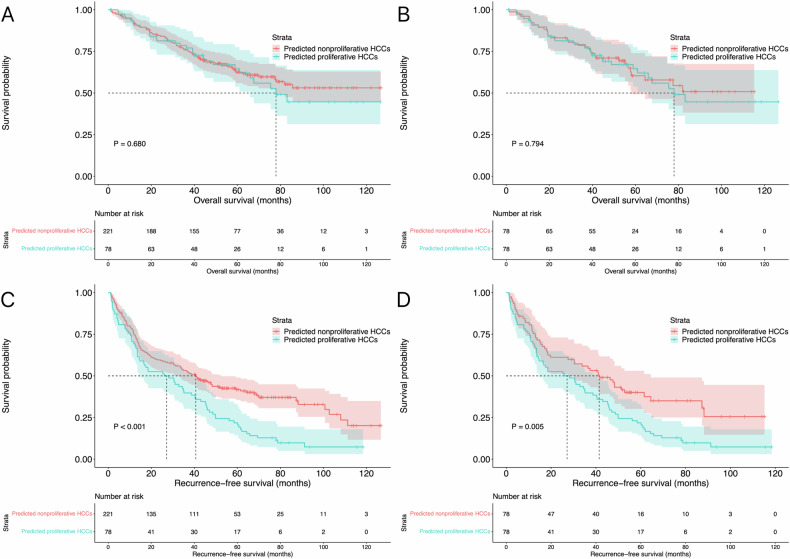
Survival curves in the subgroup of tumor size 30–50 mm. The OS of predicted nonproliferative and proliferative HCCs before matching (**A**) and after PSM (**B**) analysis. The RFS of predicted nonproliferative and proliferative HCCs before matching (**C**) and after PSM (**D**) analysis

In total, 221 and 78 patients had tumor size 30–50 mm in the predicted nonproliferative and proliferative HCCs before matching, respectively. No significant difference in OS was found between the two groups (*p* = 0.680; median OS was not reached in predicted nonproliferative HCCs vs. 77.9 months, 95% CI: 57.5–98.4 months in predicted proliferative HCCs, respectively). The median RFS was better (*p* < 0.001) in patients with predicated nonproliferative HCCs (40.7 months, 95% CI: 31.4–50.0 months) when compared to that of patients with proliferative HCCs (26.1 months, 95% CI: 11.3–40.8 months). PSM analysis was also performed in this case, and 78 pairs of patients were matched accordingly. The baseline characteristics of the patients before and after matching are summarized in Table 4. There were also no differences in OS (*p* = 0.794; median OS was not reached in predicted nonproliferative HCCs vs. 77.9 months, 95% CI: 57.5–98.4 months in predicted proliferative HCCs, respectively) between the two groups. After matching, the median RFS was also better (*p* = 0.005) in patients with predicated nonproliferative HCCs (41.5 months, 95% CI: 28.7–54.2 months) when compared to that of patients with proliferative HCCs (26.1 months, 95% CI: 11.3–40.8 months). The survival curves in the subgroup of tumor size 30–50 mm before and after matching are shown in Fig. 5.

**Table 4:** Baseline characteristics between predicted nonproliferative and proliferative HCCs in the subgroup of tumor size 30–50 mm

	Before matching			After matching		
	Nonproliferative (*n* = 221)	Proliferative (*n* = 78)	*p* value	Nonproliferative (*n* = 78)	Proliferative (*n* = 78)	*p* value
Age (years)*	54.5 ± 11.4	54.3 ± 10.7	0.886	55.7 ± 11.2	54.3 ± 10.7	0.431
Gender (*N*, %)			0.249			0.667
Male	193 (87.3)	64 (82.1)		66 (84.6)	64 (82.1)	
Female	28 (12.7)	14 (17.9)		12 (15.4)	14 (17.9)	
Liver cirrhosis (*N*, %)			0.421			0.719
Absence	15 (6.8)	3 (3.8)		5 (6.4)	3 (3.8)	
Presence	206 (93.2)	75 (96.2)		73 (93.6)	75 (96.2)	
Etiologies of hepatitis (*N*, %)			0.526			0.223
None	35 (15.8)	14 (17.9)		8 (10.3)	14 (17.9)	
HBV	166 (75.1)	60 (76.9)		62 (79.5)	60 (76.9)	
Others	20 (9.0)	4 (5.1)		8 (10.3)	4 (5.1)	
Tumor numbers (*N*, %)			–			–
Solitary	–	–		–	–	
2–3	221 (100)	78 (100)		78 (100)	78 (100)	
Neutrophil (× 10^9^, IQR)^#^	2.92 (2.17–3.72)	3.37 (2.53–4.12)	0.013	2.95 (2.32–3.85)	3.37 (2.53–4.12)	0.103
Lymphocyte (× 10^9^, IQR)^#^	1.39 (1.02–1.74)	1.28 (1.02–1.94)	0.807	1.42 (1.04–1.98)	1.28 (1.02–1.94)	0.602
Platelet (× 10^9^, IQR)^#^	134.0 (91.5–179.0)	143.0 (105.2–192.2)	0.269	127.0 (87.7–161.2)	143.0 (105.2–192.2)	0.102
Albumin (g/L, IQR)	39.5 (36.7–42.2)	39.1 (35.9–41.7)	0.462	39.6 (36.8–42.2)	39.1 (35.9–41.7)	0.466
Total bilirubin (µmol/L, IQR)	14.7 (10.4–19.5)	14.5 (10.3–20.7)	0.916	15.8 (12.9–19.5)	14.5 (10.3–20.7)	0.347

*HBV* hepatitis B virus

* Data are presented as mean ± SD

^#^ Data are presented as median and IQR

## Discussion

The results of the present study demonstrate that it is possible to noninvasively identify proliferative HCCs using the SMARS score, and predicted proliferative HCCs have a worse RFS than those of predicted nonproliferative HCCs in the entire study population as well as in the subgroup of tumor size 30–50 mm. However, the RFS does not differ between the two groups in the subgroup of tumor size smaller than 30 mm. Although proliferative HCCs typically exhibit more aggressive biological behavior, the tumor phenotype may not influence the recurrence of tumors smaller than 30 mm because achieving a sufficient ablative margin in such tumors is more straightforward than it is in larger ones [18, 19]. Nonetheless, in patients with tumor size 30–50 mm, the tumor phenotype should be considered because of the relatively high probability of insufficient ablation in addition to the more aggressive biological behavior of proliferative HCCs [20]. These results are highly significant as they may provide new insights into combination therapies. HCCs are usually derived from a background of chronic inflammation suggesting that these types of tumors could potentially be treated with immunotherapy. Proliferative HCCs usually have an immune high or intermediate status, whereas nonproliferative HCCs usually have an immune-excluded status, leading to a proportion of proliferative ones that may respond to immunotherapy [2, 4]. Therefore, combined MWA and immunotherapy may be a treatment option for patients who are suspected with proliferative phenotype. Several clinical studies using a combination of local ablation and immunotherapy have been performed [21, 22]. The results of these studies suggest that tumor phenotype should be considered when designing clinical trials.

Notably, there were no differences in OS between the predicted proliferative and nonproliferative HCCs in the entire study population as well as in the subgroups. Although proliferative HCCs demonstrate more aggressive biological behavior, they might not affect OS because the OS is a comprehensive parameter correlated with various influencing factors, such as tumor burden, liver function, and ECOG PS [23, 24]. Even though patients with predicted proliferative HCCs demonstrated a worse RFS than those with predicted nonproliferative HCCs, retreatment may still be effective in such patients [25, 26].

As described in a previous study [5], proliferative HCCs may have distinctive imaging features, including satellite lesions, lobulated tumor shapes, rim APHE, and mosaic appearance. These four imaging features have all been reported as independent risk factors of poor prognosis in patients with HCC [27–30]. Satellite lesions are a form of intrahepatic metastasis, which indicate microvascular invasion and hematogenous spread [31], and the presence of satellite lesions may lead to an early recurrence after MWA. The intrahepatic growth pattern of HCCs can be nodular, nodular with perinodular extension, confluent multinodular, massive, or diffuse in shape. In small HCCs, the tumor usually exhibits a nodular growth pattern with a round or oval shape; however, as the tumor grows larger, it shows a perinodular extension or confluent multinodular growth pattern, presenting as a lobulated shape [32]. Mosaic architecture is a marker of tumor heterogeneity [33]. As HCCs grow larger, the heterogeneity within the tumors increases, conferring a mosaic appearance and indicating a more aggressive nature. Rim APHE is an imaging feature with a targetoid appearance that favors non-HCC malignancies [34], such as intrahepatic cholangiocarcinoma, combined hepatocellular-cholangiocarcinoma, and liver metastasis of colorectal cancer. However, some HCC lesions may display rim APHE, and they usually have a worse prognosis than HCC lesions with non-rim APHE.

The current study had several limitations. First, it was a retrospective design, which might have resulted in an unavoidable selection bias, despite PSM analysis having been performed to reduce the confounding effects between the two groups. Second, the “proliferative” or “nonproliferative” HCC in the present study was not confirmed by histology and instead was designated by using the SMARS score, a predictive model. However, this is an inevitable bias since biopsy is not routinely performed before ablation. Further prospective studies are required to confirm these findings. Third, the reproducibility of the present study is limited to Eastern countries, as Western populations have different HCC etiologies compared to the Eastern.

In conclusion, the tumor phenotype should be considered by using the SMARS score before MWA, since patients with predicted proliferative HCCs have a worse RFS than nonproliferative HCCs, especially for tumors larger than 30 mm. However, the tumor phenotype may not be associated with OS.

## Data Availability

The datasets generated during and/or analyzed during the current study are available from the corresponding author upon reasonable request.

## Abbreviations

AFP: Alpha-fetoprotein
APHE: Rim arterial phase hyperenhancement
CT: Computed tomography
HCC: Hepatocellular carcinoma
IQR: Interquartile range
MWA: Microwave ablation
OS: Overall survival
PS: Performance status
PSM: Propensity score matching
RFS: Recurrence-free survival
SD: Standard deviation
